# Effects of Pregnancy and Bacterial Vaginosis on Proinflammatory Cytokine and Secretory Leukocyte Protease Inhibitor Concentrations in Vaginal Secretions

**DOI:** 10.1155/2010/385981

**Published:** 2010-08-08

**Authors:** Jennifer Balkus, Kathy Agnew, Richard Lawler, Caroline Mitchell, Jane Hitti

**Affiliations:** ^1^Department of Obstetrics and Gynecology, University of Washington, Box 356460, Seattle, WA 98195, USA; ^2^Department of Epidemiology, University of Washington, Box 359909, Seattle, WA 98195, USA; ^3^Fred Hutchinson Cancer Research Centers, Seattle, 98109 WA, USA

## Abstract

We compared vaginal proinflammatory cytokine and secretory leukocyte protease inhibitor (SLPI) concentrations among pregnant and nonpregnant women according to bacterial vaginosis (BV) status. One-hundred and twenty-two women at 12–20 weeks' gestation and 133 nonpregnant controls had vaginal concentrations of interleukin (IL)-1*β*, IL-6, IL-8, and SLPI measured by enzyme immunoassay. Multivariable linear regression was used to evaluate factors independently associated with vaginal cytokine and SLPI response. Pregnancy and BV were both independently associated with increased vaginal concentrations of IL-1*β* and IL-8; pregnant women had increased concentrations of SLPI, while women with BV had decreased SLPI concentrations.

## 1. Introduction

Ascending bacterial infection is an important antecedent of preterm birth, which continues to be a major cause of neonatal mortality. Bacterial vaginosis (BV) is a common vaginal condition that reflects a shift from the normal *Lactobacillus* predominant vaginal flora to a vaginal flora characterized by high concentrations of anaerobic bacteria. BV is consistently associated with preterm birth [1, 2]; however, antibiotic treatment trials for BV in pregnancy have not reduced preterm birth [3], leading to speculation that the maternal and/or fetal host response may be implicated in the pathophysiology of infection-associated preterm birth. We sought to evaluate the relationships between pregnancy, BV and their effects on the vaginal inflammatory response by comparing the vaginal concentrations of interleukin (IL)-1*β*, IL-6, IL-8 and the host defense molecule secretory leukocyte protease inhibitor (SLPI) among pregnant and nonpregnant women according to BV status. We hypothesized that pregnant and nonpregnant women would have similar proinflammatory cytokine and SLPI concentrations in vaginal fluid, when stratified by BV status.

## 2. Materials and Methods

We compared vaginal cytokine concentrations among women with and without BV enrolled in two separate prospective observational cohort studies. Pregnant subjects' data came from a natural history study of vaginal flora and pregnancy outcome, conducted from 2000–2004, in Seattle, Washington. Pregnant women <20 weeks' gestation, ≥16 years of age, with no history of preterm birth or major medical complications such as chronic hypertension or preexisting diabetes, were eligible to participate. Nonpregnant subjects' data came from a cohort study of racial disparities in preterm birth conducted between 2002–2006. Study participants were parous women ≥18 years of age who resided and gave birth in King County, Washington, and did not have a history of preeclampsia or hypertensive disorders. By design, the nonpregnant cohort oversampled African-American subjects. The pregnancy cohort study was approved by the University of Washington and Centers for Disease Control Institutional Review Boards. The nonpregnant cohort study was approved by the Washington State Department of Health Institutional Review Board. Written informed consent was obtained from all subjects at the time of entry into the original cohorts.

Both cohorts had identical methods for sample collection and processing. Participants were asked to abstain from vaginal intercourse for 2 days prior to their enrollment visit. Participants completed a brief questionnaire about demographics, medical history, and risk behaviors. Urine or vaginal fluid was collected for* Chlamydia trachomatis* and *Neisseria gonorrhoeae* testing by nucleic acid amplification test (Aptima Combo 2; Gen-Probe, San Diego, CA). Vaginal fluid was collected from the posterior vaginal fornix using four Dacron swabs (Puritan Hardwood Products, Guilford, Maine). One swab was rolled on a glass slide for Gram stain for BV diagnosis by Nugent criteria [4], the second was placed in culture media for *Trichomonas vaginalis* testing (InPouch TV; Biomed Diagnostics, White City, Oregon), and the third and fourth swabs were placed in cryotubes with 0.9 mL of phosphate buffered saline. These swabs were frozen at −80°C until assayed. Interleukin-1*β*, IL-6, IL-8, and SLPI were measured by enzyme linked immunosorbent assay (ELISA) at the same laboratory [5]. Acid phosphatase, a sensitive marker for semen, was detected by colorimetric method using an azo dye. Any specimens testing positive for acid phosphotase were excluded from SLPI analysis as SLPI can be found in high concentrations in seminal fluid [6, 7]. 

Subjects were included in the analysis if they had complete data for the following variables: BV status, IL-1*β*, IL-6, IL-8, SLPI, and sexually transmitted disease (STD) results (*C. trachomatis*, *N. gonorrhoeae* and *T. vaginalis*). Women with intermediate flora (Nugent score 4–6) or who tested positive for *C. trachomatis*, *N. gonorrhoeae*, or *T. vaginalis *were excluded from the analysis. Among 289 women in the pregnancy cohort with normal flora or BV, 246 women (86%) had samples available for proinflammatory cytokine testing. Of the 246 women with cytokine results, 118 (48%) women were missing one or more STD results (101 of 118 were missing *T. vaginalis* results; 98 of the 101 women had normal vaginal flora) and 6 women tested positive for an STD (4 *C. trachomatis* and 2 *T. vaginalis*). Thus 122 pregnant women, 71 with BV (Nugent score 7–10) and 51 with normal vaginal flora (Nugent score 0–3), had complete information and were included in the analysis. Among 159 women in the nonpregnant cohort with normal flora or BV, 147 women (92%) had samples available for cytokine testing. Two nonpregnant women were missing STD results and 12 women tested positive for an STD (4 *C. trachomatis* and 8 *T. vaginalis*), leaving 133 nonpregnant women, 45 with BV and 86 with normal vaginal flora in the analysis.

Chi-squared and Mann-Whitney *U* tests were used to compare demographic characteristics at baseline. The Mann-Whitney *U* test was also used for univariate analyses to compare analyte concentrations by BV and pregnancy status. Proinflammatory cytokine and SLPI values were not normally distributed and were log_10_-transformed for analysis. Mean log-transformed cytokine values and standard deviations were calculated by BV and pregnancy status. Additional analyses were conducted using multivariable linear regression to evaluate BV and pregnancy as independent factors associated with cytokine and SLPI response (adjusting for race and age). Lastly, we evaluated an interaction term for BV and pregnancy using the likelihood ratio test. This interaction term was not significantly associated with cytokine or SLPI response and was not included in the final models for each analyte. All statistical tests were assessed using a 2-sided alpha of 0.05. Analyses were conducted using Stata version 10.1 (StataCorp, Inc., College Station, TX).

## 3. Results

Nonpregnant women were older (mean age = 29.2 years (range 18–46) versus 26.7 years (range 16–43) *P* = .005), more often African-American (44% versus 20%, *P* < .001) and more likely to report ever douching (61% versus 19%, *P* < .001), and current smoking (34% versus 16%, *P* = .001) compared to pregnant women. 

In univariate analyses, pregnant women had higher concentrations of IL-1*β* (mean log-transformed values ± standard deviation: 1.94 ± 0.86 versus 1.43 ± 0.92 pg/mL; *P* < .001), IL-6 (0.63 ± 0.60 versus 0.50 ± 0.40 pg/mL; *P* = .002), and IL-8 (3.20 ± 0.76 versus 2.88 ± 0.80 pg/mL; *P* = .003) compared to nonpregnant women. Among pregnant and nonpregnant women combined, those with BV had higher concentrations of IL-1*β* (2.11 ± 0.85 versus 1.31 ± 0.83 pg/mL; *P* < .001) and IL-8 (3.15 ± 0.88 versus 2.85 ± 0.71 pg/mL; *P* = .004) compared to women without BV. Concentrations of IL-6 did not differ significantly by BV status (BV+: 0.64 ± 0.66 versus BV-: 0.50 ± 0.57 pg/mL; *P* = .09). The highest concentrations of IL-1*β* and IL-8 were found in the subset of pregnant women with BV, compared to all other subjects (IL-1*β*: 2.31 ± 0.77 versus 1.43 ± 0.87 pg/mL, *P* < .001; IL-8: 3.33 ± 0.83 versus 2.86 ± 0.76 pg/mL, *P* < .001). 

Vaginal SLPI concentrations were assessed for 109 pregnant women and 116 nonpregnant women without evidence of semen in vaginal secretions by acid phosphatase. Pregnant women had significantly higher concentrations of SLPI compared to nonpregnant women (5.08 ± 0.43 versus 4.78 ± 0.52 pg/mL; *P* < .001). Among all subjects, SLPI concentrations were lower among women with BV compared to women without BV (4.80 ± 0.53 versus 5.02 ± 0.46 pg/mL; *P* = .002). 

In multivariable analyses, pregnancy remained associated with increased concentrations of IL-8 (*P* < .02) and SLPI (*P* < .001) after adjustment for BV, race, and age (Table 1). Pregnancy was marginally associated with increased concentrations of IL-1*β* (*P* = .07) in the adjusted model. Similarly, BV remained associated with increased concentrations of IL-1*β* (*P* < .001) and IL-8 (*P* = .04), and decreased SLPI concentrations (*P* < .001), after adjustment for pregnancy status, race, and age.

## 4. Discussion

In this secondary analysis, pregnant women had increased vaginal concentrations of the proinflammatory cytokine IL-8 as well as the mucosal host defense molecule SLPI, regardless of BV and other cofactors. In addition, we confirmed others' findings that BV, regardless of pregnancy status, is accompanied by increased vaginal proinflammatory cytokine concentrations along with decreased SLPI concentrations [8–10]. 

The primary limitation of this report is that it is a secondary analysis of two different study populations. A second limitation is that missing data in the pregnancy cohort required us to exclude a number of women from the analysis. Much of the missing data was missing by design; therefore, the exclusion of women with data missing by design should not bias the results.

Taken together, these data suggest that BV should be regarded as a proinflammatory condition for pregnant and nonpregnant women. The vaginal inflammatory response may be “on alert” in pregnancy, and antigenic stimulation (such as BV) appears to result in a more exuberant inflammatory response among pregnant, compared to nonpregnant women. Moreover, we observed that SLPI concentrations are greater in pregnant women compared to their nonpregnant counterparts, regardless of BV status. Increased SLPI in the context of pregnancy may be a physiologic host defense mechanism to protect against ascending infections that could result in preterm birth. Further prospective studies of vaginal immunology are needed to help elucidate the effects of pregnancy, vaginal flora and other factors on lower genital tract host defenses and the implications for reproductive health.

## Figures and Tables

**Table 1:** Linear regression models* for the effect of pregnancy and bacterial vaginosis on log-transformed vaginal cytokine and SLPI concentrations.

	Coefficient	95% Confidence Interval	*P*-value
Interleukin-1*β*			
Pregnancy	0.199	(−0.019 to 0.416)	.07
BV	0.763	(0.542 to 0.984)	<.001

Interleukin-6			
Pregnancy	0.025	(−0.139 to 0.189)	.76
BV	0.125	(−0.041 to 0.291)	.14

Interleukin-8			
Pregnancy	0.255	(0.048 to 0.462)	.02
BV	0.216	(0.006 to 0.426)	.04

SLPI**			
Pregnancy	0.408	(0.280 to 0.537)	<.001
BV	−0.397	(−0.529 to −0.266)	<.001

*Models contain the analyte of interest as the dependent variable and covariates for BV, pregnancy, race (as an indicator variable), and age (as a continuous variable). **Restricted to women who were acid phosphatase negative.

## References

[1] Kurki T, Sivonen A, Renkonen O-V, Savia E, Ylikorkala O (1992). Bacterial vaginosis in early pregnancy and pregnancy outcome. *Obstetrics and Gynecology*.

[2] Hillier SL, Nugent RP, Eschenbach DA (1995). Association between bacterial vaginosis and preterm delivery of a low-birth-weight infant. *New England Journal of Medicine*.

[3] McDonald HM, Brocklehurst P, Gordon A (2007). Antibiotics for treating bacterial vaginosis in pregnancy. *Cochrane Database of Systematic Reviews*.

[4] Nugent RP, Krohn MA, Hillier SL (1991). Reliability of diagnosing bacterial vaginosis is improved by a standardized method of gram stain interpretation. *Journal of Clinical Microbiology*.

[5] Williams MA, Mahomed K, Farrand A (1998). Plasma tumor necrosis factor-*α* soluble receptor p55 (sTNFp55) concentrations in eclamptic, preeclamptic and normotensive pregnant Zimbabwean women. *Journal of Reproductive Immunology*.

[6] Ohlsson K, Bjartell A, Lilja H (1995). Secretory leucocyte protease inhibitor in the male genital tract: PSA- induced proteolytic processing in human semen and tissue localization. *Journal of Andrology*.

[7] Agnew KJ, Aura J, Nunez N (2008). Effect of semen on vaginal fluid cytokines and secretory leukocyte protease inhibitor. *Infectious Diseases in Obstetrics and Gynecology*.

[8] Beigi RH, Yudin MH, Cosentino L, Meyn LA, Hillier SL (2007). Cytokines, pregnancy, and bacterial vaginosis: comparison of levels of cervical cytokines in pregnant and nonpregnant women with bacterial vaginosis. *Journal of Infectious Diseases*.

[9] Draper DL, Landers DV, Krohn MA, Hillier SL, Wiesenfeld HC, Heine RP (2000). Levels of vaginal secretory leukocyte protease inhibitor are decreased in women with lower reproductive tract infections. *American Journal of Obstetrics and Gynecology*.

[10] Novak RM, Donoval BA, Graham PJ (2007). Cervicovaginal levels of lactoferrin, secretory leukocyte protease inhibitor, and RANTES and the effects of coexisting vaginoses in Human Immunodeficiency Virus (HIV)-seronegative women with a high risk of heterosexual acquisition of HIV infection. *Clinical and Vaccine Immunology*.

